# Optimal Transport for Free Energy Estimation

**DOI:** 10.1021/acs.jpclett.2c03523

**Published:** 2023-02-07

**Authors:** S. Decherchi, A. Cavalli

**Affiliations:** Fondazione Istituto Italiano di Tecnologia, Via Morego 30, Genoa 16163, Italy

## Abstract

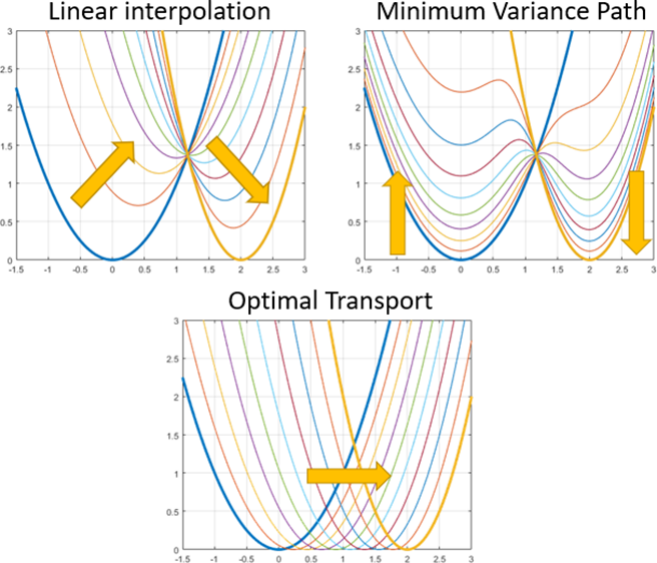

Optimal transport theory is a growing field of mathematics, which has recently found many applications. Here we take advantage of optimal transport for computational free energy estimation. We show analytically, and then via simulation, that this approach is effective in terms of optimizing the barriers of an alchemical transformation.

Computing free energy differences is a classical^1^ and not trivial task in many physical and chemical applications, including surface science, nanomaterials, and drug discovery.^2^ Previous research has established remarkable connections between thermodynamics and optimal transport theory,^3−6^ in particular showing that the optimal transport approach minimizes the production of entropy when continuously transforming one distribution into another. Here we show how optimal transport theory can be used to optimize the barriers (kinetics) of paths used to mix the Hamiltonians in free energy computations. This leads to the definition of a potential function we call Wasserstein potential. Hereafter, we first introduce the optimal transport approach and then build a formal link between it and free energy estimations via equilibrium methods. We demonstrate its efficacy analytically for harmonic oscillators and then test the method on a one-dimensional simulation. Finally, we report on some practical considerations, perspectives, and conclusions.

Optimal transport^7^ is a powerful and effective theory that maps two distributions with the minimal effort. Say *X* is the  space where the mass lives and *p*_1_(*x*) the associated starting mass distribution, we want to transform this mass into distribution *p*_2_(*x*) in the same space. Assume also the existence of a not degenerate cost function *c*(*x*, *y*), which measures the cost of transporting the mass from point *x* to point *y*. Then, we define a transport plan γ(*x*, *y*) as the function that gives the amount of mass in *x* that we want to move in *y*. Mass conservation leads to
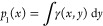
1
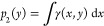
2

From the distribution viewpoint, this means that γ(*x*, *y*) is the joint probability distribution of *p*_1_(*x*) and *p*_2_(*x*). Also, the infinitesimal mass moved from *x* to *y* is hence γ(*x*, *y*) d*x* d*y* and the associated transport cost is *c*(*x*, *y*)γ(*x*, *y*) d*x* d*y*. The aim of optimal transport is to overall minimize this transportation cost with respect to γ(*x*, *y*) using distance function *c*(*x*, *y*). In other words, one wants
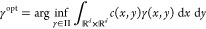
3where Π is the whole set of joint distributions whose marginals are *p*_1_(*x*) and *p*_2_(*x*) and γ^opt^ is the optimal transport plan. If as a cost function a *p*-norm is used, this gives rise to the Wasserstein distance:
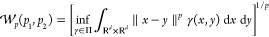
4

This distance definition has been applied to several domains from image processing^8^ to generative learning in deep learning^9^ with wide success. It can be shown^10^ that, for instance, for the specific case of Gaussian distributions and L2-norm, one can obtain analytically the geodesic distribution *p*_*m*_(*x*; λ), which is *p*_1_(*x*) for λ = 0 and *p*_2_(*x*) for λ = 1. Setting  and , one can show^10^ that , where
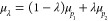
5and
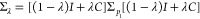
6where , the *I* symbol is the identity matrix, and the inversion can be replaced by pseudoinversion in the case of a singular or close to singular covariance matrix. In general, when the distribution is known by sampling or is not a Gaussian, several methods, chiefly the Sinkhorn algorithm,^11^ are available to numerically estimate the Wasserstein distance.

We have so far reported that optimal transport theory naturally leads to a notion of distance between distributions and such a notion captures well the effort in morphing them. Now, the point of this paper is that morphing distributions is not only the task of optimal transport but also the essence of alchemical methods for free energy estimation. By alchemical we mean that the transformation does not involve a physical path.

In free energy estimation via alchemical methods, one is endowed with two Hamiltonians,  and , corresponding potentials *U*_1_ and *U*_2_, and two configurational Boltzmann distributions, *p*_1_(*x*) and *p*_2_(*x*). One desires to obtain free energy difference Δ*F* between the two states. We hypothesize here to have the canonical ensemble, and hence, we will not discuss kinetic energy terms as they factorize the partition function.

As there could be no shared configuration space overlap, one usually builds a path to connect the two states and splits the space in a series of intermediate windows to allow superimposition of the distributions and help the convergence of free energy reconstruction methods such as thermodynamic integration,^12^ the Zwanzig formula,^13^ and the Bennett acceptance ratio.^14^ It is customary when performing free energy computations to define a parameter λ ∈ [0, 1] that rules the morphing of the potentials. Apart from cases in which there may be numerical instabilities, the natural choice for this task is to obtain the convex combination of the potentials to define the mixed potential *U*_m_ of each simulation window:
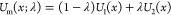
7

However, one should remember that the real goal is mixing probability distributions, not the potentials. If one applies an optimal transport approach here, the prescription is rather different. The intermediate distributions should live along the geodesic path that connects the two configurational Boltzmann distributions *p*_1_(*x*) and *p*_2_(*x*). Hence, we propose to use as intermediate distributions those that can be obtained as Wasserstein barycenters.^15^ A Wasserstein barycenter for two distributions is a new distribution defined by
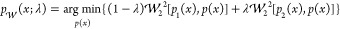
8where  indicates a squared Wasserstein distance using a L2-norm. Note that the extension to multiple end point distributions^15^ is straightforward. This leads to the introduction of the corresponding Wasserstein potential:
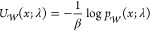
9

Here too, for λ = 0 and λ = 1, one correctly recovers the initial and final systems, respectively; obviously, one can always adjust this potential by an offset to align it to a reference potential. Nevertheless, the intermediate distributions and hence potentials are usually very different from the convex combination commonly utilized. In general, the usual convex combination (linear interpolation) of potentials and the convex combination of the not normalized distributions (i.e., the minimum variance path^16−18^) are not equal to an optimal transport path, rendering this new potential definition significantly different from previous approaches. In detail, the minimum variance path (MVP) introduced by Blondel^16^ is defined by
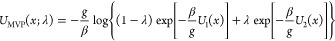
10where *g* can vary depending on the derivation, being equal to 2 in the first proof.^16^ This mixing potential, apart from the constant *g*, is the convex combination of the un-normalized Boltzmann distributions and is significantly different from . MVP is proven to minimize the mean squared estimation error (MSE) yet gives no warranties about the nature of the intermediate potentials along the path. Interestingly, the MVP potential is equivalent to the envelope distribution sampling (EDS) method (up to a constant)^19^ for *g* = 1 and using λ = 0.5 only.

We can check the effect of using the classical λ schedule done via a convex combination of potentials versus that induced by optimal transport. We restrict ourselves to the simple but instructive and analytical case of two Gaussian one-dimensional distributions (two harmonic oscillators), which differ only in the position of the minima and whose potential values is the same; this situation mimics the most classical chemical interaction, namely the chemical bond between particles. The free energy difference of these two systems is analytically 0. To compute the free energy, we evaluate the thermodynamic integration integral:
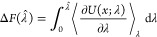
11and we check the behavior of the integrand and of .

In the following, the two oscillators are , where , and . The two schedules, namely, the convex combination and the optimally transported one, are defined by the two mixing potentials. The usual one will be indicated as *U*_m_:
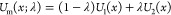
12whereas it is easy to show from Delon and Desolneux^10^ that
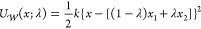
13obviously at the end points λ = 0 and λ = 1 they both attain the values of *U*_1_(*x*) and *U*_2_(*x*). In the linear interpolation case, we weaken the springs and the particle is attached to both springs during the simulation; in optimal transport, we directly move the reference center. At no time is the particle attached to both reference points (see Figure 1). Interestingly, even if we started from an alchemical viewpoint, the prescription of the optimal transport is to use a potential that physically moves the center from the starting point to the destination. Hence, an optimal alchemical path leads naturally to the interpolation of parameters or even to umbrella sampling^20^ for this case. Now we quantify analytically the differences between the two strategies.

**Figure 1:**
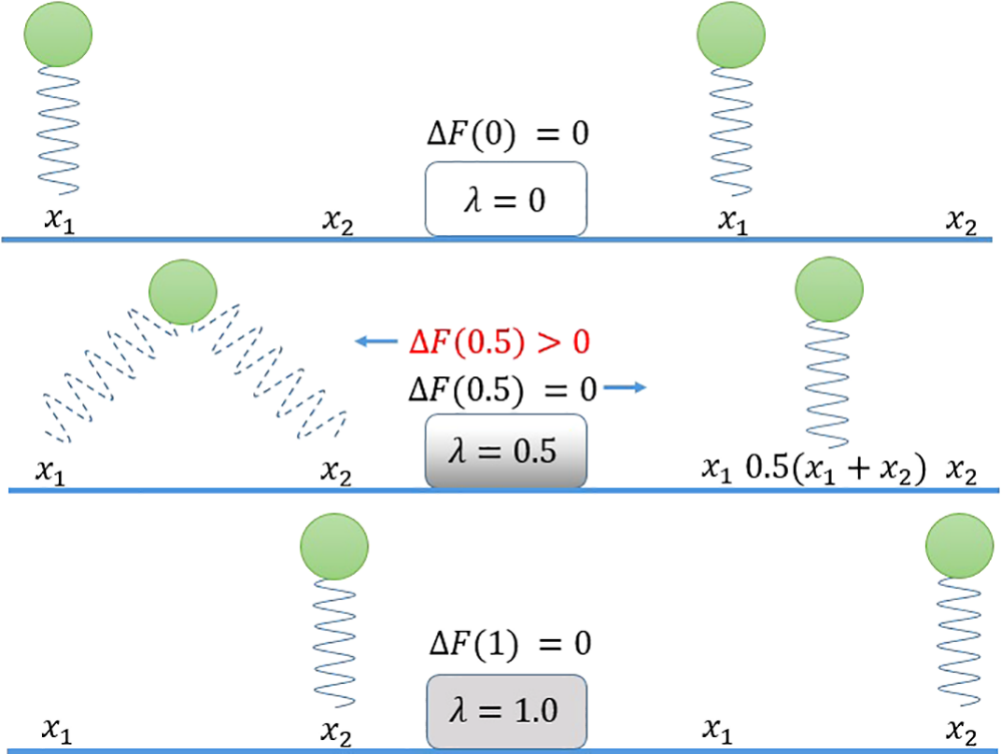
Linear interpolation of the potentials (left) vs optimal transport (right). The optimal transport prescription leads to a physical path whose integrand in thermodynamic integration is identically null.

Here, we use thermodynamic integration to estimate the free energy difference. To proceed, we need to compute the derivative of the mixed Hamiltonian. For the sake of simplicity and without a loss of generality, we will assume a dimensionless potential and a temperature coefficient of 1. First, we analyze the usual convex combination of the potentials. The derivative of the Hamiltonian is
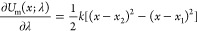
14

We need then to evaluate the bracket that is the expectation of this quantity:
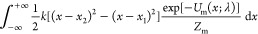
15

The configurational partition function *Z*_m_ value can be evaluated analytically and is
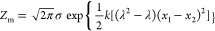
16

The integral (excluding the partition function) can also be evaluated analytically, and one finds that  is equal to
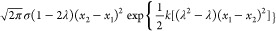
17collecting and simplifying one finds that collectively the integrand of thermodynamic integration is
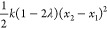
18

Then, as expected
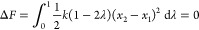
19

It is interesting now to evaluate what happens for λ = 0.5 as the peak of the transformation is located here:
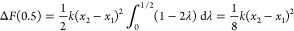
20

Notably, this stress is proportional to the spring constant of the harmonic oscillator and also to the gap between the two means. That is, in the worst case of two very different *x*_1_ and *x*_2_ values, this value can become arbitrarily large; in other words, the thermodynamic integration integrand can lead to an arbitrarily steep linear function in λ despite the analytical value of the integral being 0.

We can now turn our attention to what happens when the potential induced by the optimal transport is used, that is using . In this case, the derivative is
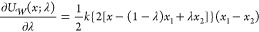
21that is
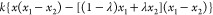
22

At a given λ value, we can then evaluate the expectation (the bracket) of this expression. First, one should note that (1 – λ)*x*_1_ + *λx*_2_ is the center of potential along the λ schedule. Hence, if we take the expectation *x*(*x*_1_ – *x*_2_), then, up to a multiplicative factor due to the partition function, this gives exactly [(1 – λ)*x*_1_ + *λx*_2_](*x*_1_ – *x*_2_). This cancels the second term (which is constant with respect to the expectation operator), hence giving an integrand always equal to zero. We have thus shown that under the optimal transport schedule, we obviously correctly recover 0 as the free energy difference and more importantly find that the integrand is identically 0 for any value of λ. This is the best possible integration path, hence a minimum free energy path in alchemical space. This result can also be proven using the Zwanzig and Bennett estimators (not shown for the sake of brevity).

In the specific case of harmonic oscillators with the same spring constant, however, one can note that the mixed distribution induced by the linear interpolation is indeed identical to that from optimal transport. The reason why one obtains different barriers is the unwanted shift in the potentials that is produced by the linear interpolation (see Figure 2). Indeed, in this specific case, it is easy to show that the mixed linear interpolation potential can be rewritten as
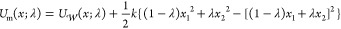
23

**Figure 2:**
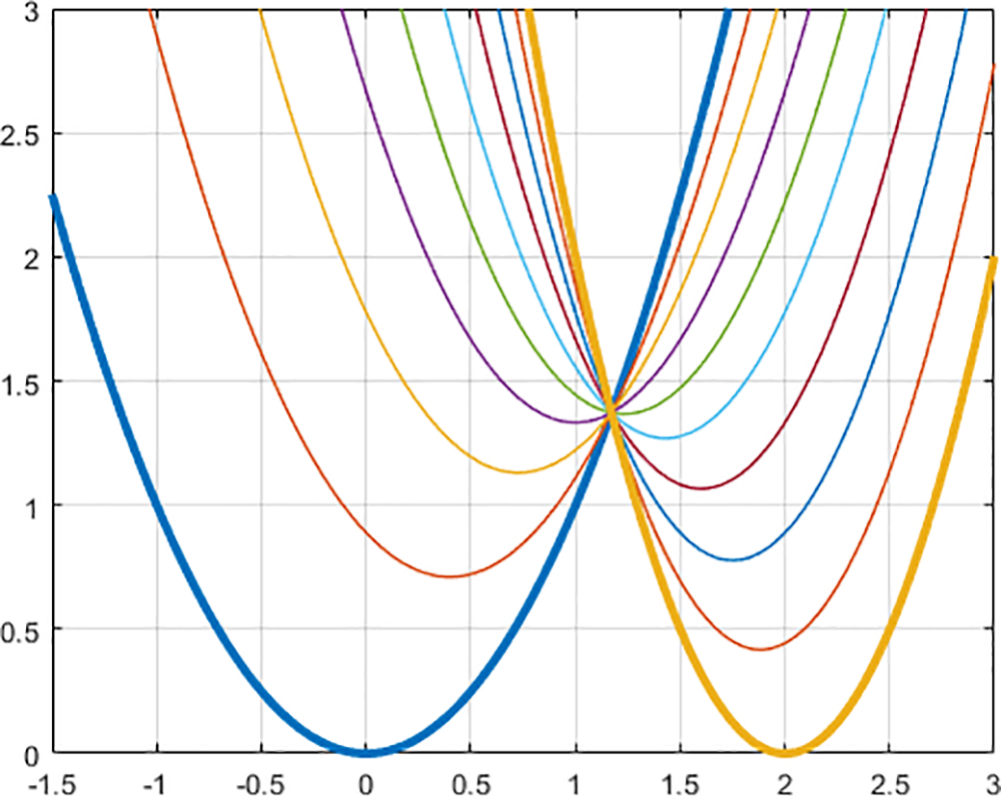
Under linear interpolation, the mixing potentials suffer from an undesirable shift. The thick line shows the reference harmonic potentials.

Note that this behavior is a very peculiar feature to Gaussian distributions with the same spring constant. When the spring constants are different, by leveraging Gaussian distribution properties one can write *U*_m_(*x*; λ), up to an arbitrary offset as
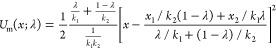
24from which one can match the center or the spring constant of  (we now call the parameter *t* to underline the fact that we use it as the degree of freedom to allow the spring constant matching). However, matching the mean will mismatch the spring constant and vice versa. Hence, one in this case can say that the two potentials are equivalent but not identical (unless a further shift in the *x* coordinates occurs when spring constant matching is done). In general, indeed, for non-Gaussian distributions this easy equivalence between  and *U*_m_(*x*; λ) is lost. Despite this state of affairs, fixing the offset of the linear interpolation is beneficial and suggests a correction. Indeed, for each λ value, one can shift the minimum value of mixing potential *U*_m_(*x*; λ) to the value *E*(λ) = (1 – λ) min_*x*_*U*_1_(*x*) + λ min_*x*_*U*_2_(*x*). In other words, we correct for the nonlinear behavior of the min operator by substituting the observed minimum value with the one coming from the convex combination of the minima. Hence, the corrected linear interpolation potential is
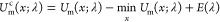
25

Interestingly, it is easy to show that this potential is equal to the λ-EDS^19^ method in the limit of the, there specified, *s* parameter that goes to 0 and for the here proposed choice of the potential offsets. Also, the same adjustment can be applied to the Wasserstein potential when the two reference minimum values, min_*x*_*U*_1_ and min_*x*_*U*_2_, differ; indeed, the benefits of the Wasserstein potential are related to the shape of only the potential.

Now we define an illustrative simulative algorithm to apply the optimal transport method mentioned above to arbitrary end point distributions. At this stage, the protocol is not trivially applicable to high-dimensional distributions, yet it allows one to apply the principle to a simulative case. We define the following protocol:

1. Sample, via plain MD, from the starting *U*_1_ and ending potential functions *U*_2_.
2. Build histograms of the two distributions, and for each λ value, compute numerically the optimally transported discrete barycenter via eq 8.
3. Obtain the Wasserstein potential (eq 9) for each λ.
4. Train a neural network for each λ such that one has an out-of-sample  potential function. This step is needed to have an interpolative model for .
5. Use the above learned potentials to run the usual sampling, and use the Bennett acceptance ratio to estimate the free energy difference at each λ window. Summing the contributions of all windows estimates the desired free energy difference.

In general, computing the Wasserstein distance is a computationally complex task; hence, we restrict this process to a monodimensional case. In detail, to estimate the Wasserstein barycenter (eq 8), we take advantage of the entropic regularized form of the Wasserstein distance,^15^ known as the Sinkhorn algorithm. As regularization (we always used a value of 0.03) can have a negative impact on the quality of the obtained barycenter, we use a posteriori a debiasing method to improve the solution quality.^21^ The procedure mentioned above can be easily performed using the PythonOT library^22^ and the function *bregman.barycenter_debiased()*, which we used in this experiment.

As one needs to sample from this potential to run BAR, a feasible approach for solving this problem is to approximate the potential via a neural network. This approach is simple and flexible and allows one to learn any potential function due the universality of these approximators. In this example, we take advantage of a shallow neural network, namely the kernel -regularized least-squares method.^23^ We represent the potential with the following linear combinations of Gaussian basis functions:
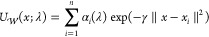
26where *n* is the number of training points (histogram bins), *x*_*i*_ terms are the corresponding centers, α_*i*_(λ) terms are coefficients that are learned starting from the discrete potential (and hence depend on λ), and γ is a scale factor and here is taken to be 1.0/(2*s*^2^), where *s* is the bin size. To obtain the optimal coefficients α_*i*_, one minimizes a regularized least-squares cost function:^23^
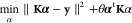
27where **K** is the *n* × *n* kernel matrix (Gaussian basis), **y** is the vector where , and γ is a scale factor and is taken to be 1.0/(2*s*^2^), where *s* is the bin size. Lastly, θ is a regularization coefficient we set to 10^–5^, which stabilizes the system solution and gives a smooth potential. The optimal **α** vector is hence the solution of the following system:
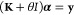
28

Once the neural Wasserstein potentials are available, one can proceed as usual in estimating the free energy, which is running one simulation for each λ; this time, however, for each λ we use the neural potential  to run the simulation except for the end points λ = 0 and 1 where we use the original potential functions.

To test this proof of principle protocol, we compare it to the linear, usual λ energy interpolations, the MVP method (*g* = 1 in the non-normalized probability density combination), and linear interpolation in which we correct for the mismatch of the minima (which we have shown previously helps in reducing barriers). The test system is a harmonic oscillator versus a shifted double-well potential. The double-well potential is zero centered, whereas the harmonic oscillator is shifted of 0.5 nm; hence, the systems do not bear configurational superposition. The double-well potential is represented by the equation
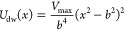
29where *b* = 0.1 and *V*_max_ = 1.0 kJ/mol. The harmonic oscillator *U*_h_(*x*) is centered at *x*_0_ = 0.5 nm, and the oscillator frequency is set to ν = 2.0 ps^–1^; hence, the spring constant is 157.91 kJ/mol. Mass is always unitary. This test can be considered a prototypical case of two systems in which there is no configurational overlap; hence, performing a perturbation from one Hamiltonian to another can be difficult.

To run the simulations, we used the *NVT* ensemble with no periodic boundary conditions, Langevin dynamics, and the Velocity–Verlet integrator.^24^ The MD engine, the neural network, and the Bennett acceptance ratio are all developed in house in python and JAX.^25^ To compute forces, we obtain analytically the gradient via JAX automatic differentiation. We used 21 equally spaced λ values, a time step of 10 fs, and a temperature of 300 K. We run 2 ns of plain MD for harmonic system *U*_h_ and double-well *U*_dw_ to sample them and build the histograms needed to train the neural networks. Then we used the protocol mentioned above running up to 10 ns for each intermediate potential. In Figure 3, there is a representation of the learned Wasserstein potentials. We repeated the full calculations three times to estimate the standard deviation; the confidence level of the uncertainties is 95% and is computed according to the *t*-Student distribution with two degrees of freedom as . We checked the convergence of the free energy value and the capability of avoiding large barriers along the alchemical path. Figure 4 shows the convergence versus the number of samples. Confirming previous experiments,^19^ we found the MVP final estimate (orange line) is both the least accurate and precise (−0.34 ± 0.20 kJ/mol) despite the fact theory dictates a minimization of the MSE, whereas energy interpolation and optimal transport are both accurate and precise. Linear interpolation for the longest simulation and for the final free energy gives a value of −0.40 ± 0.04 kJ/mol, whereas optimal transport affords a value of −0.40 ± 0.00 kJ/mol. These effects are more pronounced for shorter sampling times. This result confirms that despite the rigorous derivation, practically using the MVP approach can be problematic. Also, optimal transport gives smaller deviations particularly in the small sample regime. The second aspect, and more central to this discussion, we evaluated is the capability of avoiding large barriers during the transformation. In Figure 5, we draw the free energy difference along the path against the λ value. The results show that the linear path (green line) explores free energy differences very distant from the target one; MVP (orange) has a much weaker effect in this regard, yet it bears kinetic barriers. The optimal transport solution (blue) shows an almost flat line, which again confirms the efficacy of the approach. Lastly, linear interpolation with the corrected minima (red) has a much improved barrier with respect to the plain linear interpolation counterpart yet is not on par with optimal transport. We can hypothesize that at a practical level, minimizing the barriers (as noted in a nonequilibrium setting^26^) is more effective than minimizing the MSE per se.

**Figure 3:**
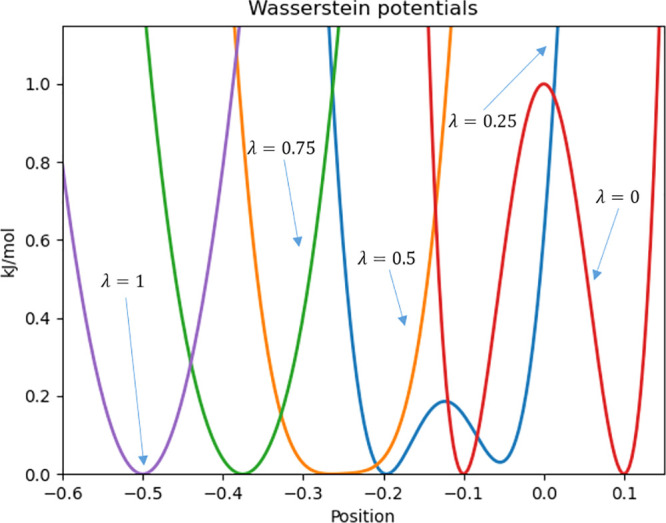
Example Wasserstein potentials that morph a double-well (λ = 0) into a harmonic (λ = 1) oscillator.

**Figure 4:**
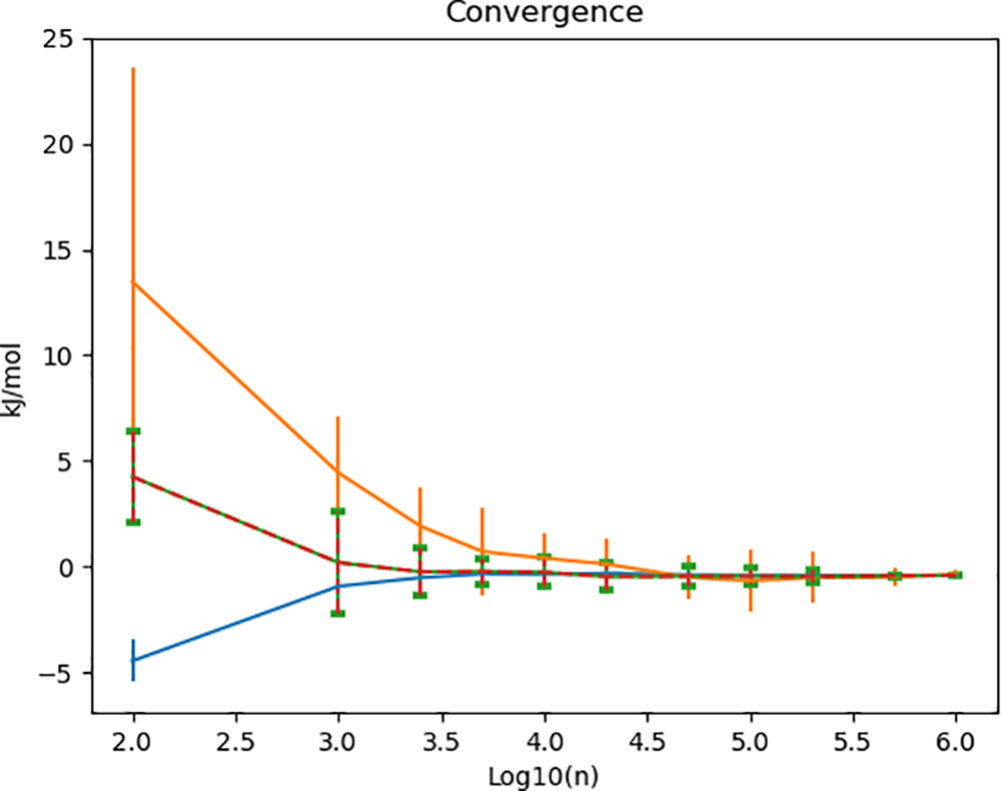
Convergence of the morphing schedules. The *x* axis shows the log number of time steps, and the *y* axis the free energy difference. Linear interpolation in green (with thicker errors bars), linear interpolation with corrected minima in red (dashed line), MVP in orange, and optimal transport in blue.

**Figure 5:**
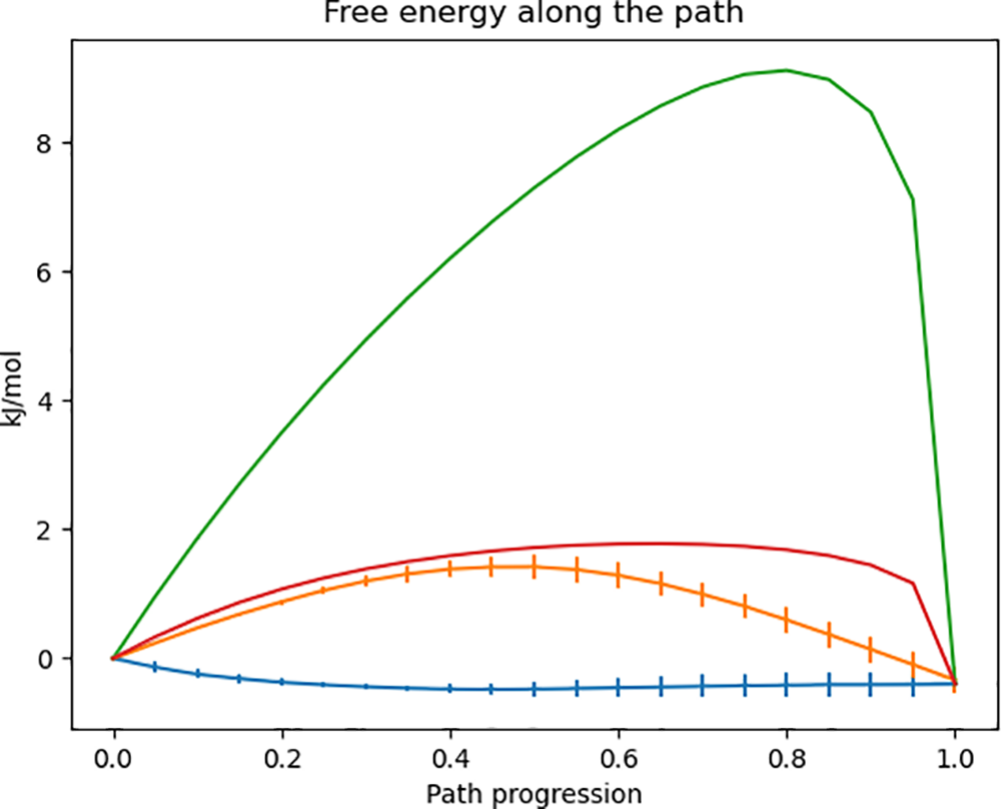
Free energy difference of the morphing schedules along the progression of the transformation. The *x* axis shows the λ index, and the *y* axis the free energy difference with error bars. Linear interpolation in green, MVP in orange, optimal transport in blue, and corrected linear interpolation in red.

To further check and strengthen these findings, and similarly to a benchmark approach,^27^ we simulated again the same potentials but this time employing two and four non-interacting particles. In other words, the new harmonic system is
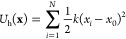
30and the double-well is
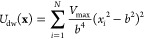
31where *N* is the number of particles and **x** is the particle position vector. We summarize in Table 1 our findings and report the final free energy values and the associated uncertainties. Note that, as the particles are non-interacting, the free energy difference for *N* = 2 is just twice that for *N* = 1 and so on. Hence, assuming −0.40 kJ/mol to be a perfectly converged result for *N* = 1, for *N* = 2 one could expect that the exact free energy is −0.80 kJ/mol and for *N* = 4 one should expect a value of −1.60 kJ/mol. Overall, we found that OT always exhibits a much higher precision, convergence is reached quicker than linear interpolation, and the estimate is also the most accurate. On the contrary, MVP is systematically the least precise and accurate and has significant convergence difficulties (very slow) for an increasing number of particles, confirming previous results.^19^

**Table 1:** Comparison of the Minimum Variance Path (MVP), Optimal Transport (OT), Linear Interpolation, and Linear Interpolation with Correction for Minima (linearC)^a^

*N*	Δ*t* (ns)	linear	linearC	MVP	OT	converged
1	0.1	–0.25 ± 0.69	–0.25 ± 0.69	0.41 ± 1.20	–0.34 ± 0.07	–0.40
1	0.2	–0.46 ± 0.66	–0.46 ± 0.66	0.14 ± 1.18	–0.30 ± 0.09	–0.40
1	0.5	–0.43 ± 0.48	–0.43 ± 0.48	–0.48 ± 1.02	–0.37 ± 0.02	–0.40
1	1	–0.44 ± 0.41	–0.44 ± 0.41	–0.66 ± 1.49	–0.40 ± 0.09	–0.40
1	5	–0.41 ± 0.04	–0.41 ± 0.04	–0.47 ± 0.41	–0.40 ± 0.04	–0.40
1	10	–0.40 ± 0.04	–0.40 ± 0.04	–0.34 ± 0.20	–0.40 ± 0.00	–0.40
2	0.1	0.01 ± 0.54	0.01 ± 0.54	6.27 ± 1.25	–0.98 ± 0.23	–0.80
2	0.2	–0.08 ± 0.52	–0.08 ± 0.52	4.40 ± 1.62	–0.82 ± 0.09	–0.80
2	0.5	–0.58 ± 0.86	–0.58 ± 0.86	1.70 ± 1.14	–0.84 ± 0.02	–0.80
2	1	–0.48 ± 0.53	–0.48 ± 0.53	1.32 ± 1.02	–0.78 ± 0.06	–0.80
2	5	–0.77 ± 0.37	–0.77 ± 0.37	–0.28 ± 0.31	–0.78 ± 0.02	–0.80
2	10	–0.80 ± 0.23	–0.80 ± 0.23	–0.69 ± 1.51	–0.79 ± 0.03	–0.80
4	0.1	0.87 ± 1.09	0.85 ± 1.14	35.07 ± 4.55	–1.85 ± 0.43	–1.60
4	0.2	–0.35 ± 0.45	–0.36 ± 0.42	33.65 ± 2.24	–1.73 ± 0.01	–1.60
4	0.5	–1.34 ± 1.12	–1.34 ± 1.11	32.96 ± 0.63	–1.56 ± 0.06	–1.60
4	1	–1.24 ± 0.24	–1.25 ± 0.23	27.69 ± 15.5	–1.57 ± 0.14	–1.60
4	5	–1.57 ± 0.35	–1.57 ± 0.35	14.90 ± 4.16	–1.60 ± 0.03	–1.60
4	10	–1.56 ± 0.25	–1.56 ± 0.25	11.40 ± 3.13	–1.59 ± 0.01	–1.60

a *N* is the number of particles. Δ*t* is the simulation time for each λ, and the free energy difference is in kilojoules per /mole. The confidence level of the uncertainties is 95%.

In this work, we have introduced optimal transport theory as a tool for equilibrium free energy estimation. The shortcomings of linear interpolation of the potentials and of MVP are well-known and have been extensively reported in the literature. The approach presented here provides a mathematical solution that is particularly effective when there is no configurational overimposition. Conventional Hamiltonian morphing methods may tend to destroy and rebuild distributions, whereas optimal transport tends to more gently move and adapt probability mass. This pathology is particularly true for MVP; its noncompliance with optimal transport leads to large barriers or hysteresis. The optimal transport gentleness, however, is not for free. To build these smooth paths, one needs a certain degree of knowledge of the starting and ending distributions; this is the histogram step in the proposed protocol. To what extent this knowledge is needed is not yet clear, particularly for high-dimensional spaces. Additionally, performing optimal transport in high dimensions is far from trivial and is the subject of intense research.

In a first approximation, one can try to devise rules from this general optimal prescription. Something that is evident from this study is that optimal transport naturally leads to the interpolation of parameters. Another observation is that the alignment of distributions (in configurational space) can help before any morphing. This derived prescription confirms indirectly the validity of the Frankel and Ladd method^28^ and later developments by Cecchini and co-workers.^29^ Also, linear interpolation can benefit from alignment of values of minima; the same techinique could be used to align Wasserstein potentials when the values of the end point potential minima differ.

We mention that other formulations based on the Fokker–Planck equation taking advantage of optimal transport^3−6,30,31^ are possible leading to a nonequilibrium setting. Our observations also relate to targeted free energy^32^ perturbation (FEP) where one supposes having an invertible map between end states to facilitate the free energy estimation convergence. Optimal transport delivers such an optimal map in its most difficult formulation, namely the Monge one.^7^ In our approach, we do not search for the Monge map, which is an even more difficult problem; we just work with the Kantorovich relaxation further made computationally efficient via entropic regularization^11^ to obtain a numerical approximation of the intermediate distributions. Recent computationally scalable attempts to find an approximate map for targeted FEP^33−35^ take advantage of normalizing flows, yet they were not linked with optimal transport as in these works only the map is used and its optimality is not discussed. The optimal transport-inspired protocol presented here is not immediately amenable to high-dimensional spaces, yet one can try to retain as much as possible the good features of the approach. We can envision that using optimal transport as a regularization operator may lead to improved and fast converging normalizing flows for targeted FEP. Such an approach has been pioneered in machine learning^36^ and could be tried in computational chemistry.

In conclusion, we have presented herein a method for the morphing of two Hamiltonians that bear interesting properties. We expect optimal transport to be, at least in principle, a valuable asset for computational free energy estimations.

## Data Availability

The data and code that support the findings of this study are available from the corresponding author upon request.

## Supporting Information Available

Transparent Peer Review report available (PDF)
