# Supplementary material for: Optimal Transport for Free Energy Estimation
Source: J Phys Chem Lett. 2023 Feb 7;14(6):1618–25. doi: 10.1021/acs.jpclett.2c03523 (PMC9940200; doi:10.1021/acs.jpclett.2c03523)
Supplement: Supplementary file 1 — jz2c03523_si_001.pdf [file jz2c03523_si_001.pdf]

jz-2022-035234.R1

Name: Peer Review Information for "Optimal Transport for Free Energy Estimation"

First Round of Reviewer Comments

Reviewer: 1

Comments to the Author

This manuscript describes optimal transport as a protocol for defining a path between two states of a system for the purpose of computing their free energy difference.

The first third of the manuscript lays out the basic ideas and defines relevant quantities. I have the following minor suggestions to improve the clarity of this part

1. Near Eq. 3, C is defined as minimizing the cost "with respect to c(x,y)". The quantity being varied to find the minimum is gamma(x,y), so my understanding of "with respect to" in this context would be gamma, not c. I could see that "with respect to" might mean "based on the definition using", and arguably this is not incorrect usage. I think however it is confusing and I suggest the authors reconsider the phrasing of the statement if this is intended. Or perhaps they actually meant "with respect to gamma(x,y)", and this is just a typo.

2. The p appearing both as a superscript and subscript on W in Eq. 4 is confusing. It took me some time to understand that the superscript is an exponent, and the subscript indicates that it is a p-norm. I suggest putting the W and its arguments in brackets, with the p superscript on that, or putting brackets on the right-hand side with a 1/p exponent.

3. After Eq. 4, it is stated that "one can obtain analytically the geodesic distribution". The context suggests this appears to be a consequence of the Wasserstein distance, but I don't see the connection. What procedure does one perform to obtain the geodesic distribution? Is it gamma for the minimum when p1 and p2 are Gaussians? (apparently not, as it doesn't depend on y) Presumably, I could consult the reference for clarification, but I should be able to understand the statement by itself (apart from its derivation) without consulting another paper.

4. If Eq. 8 is defining B, the barycenter, I suggest putting "B \equiv" on the left-hand side, so it is an equation. This makes it easier to understand when looking back at it from later in the paper.

The middle third of the paper treats a toy model, characterizing how the methods under consideration would perform when computing the free energy difference using thermodynamic integration (TI). This part of the paper misses the mark, and does not provide support for the value of the optimal transport method.

The performance of any free-energy calculation is concerned separately with the accuracy and the precision of the calculation. Regarding accuracy, assuming the system is well-sampled for each lambda

(which is not an issue in this work), then the main determinant of accuracy is the curviness of the integrand -- more integration points are needed to navigate accurately where the integrand is not linear. An integrand the depends strictly linearly on lambda will have absolutely no difficulty yielding an accurate result (just two quadrature points is sufficient). Both optimal transport and the convex combination of potentials (CCoP) have integrands that depend linearly on lambda. It does not matter that one is horizontal and the other is not -- neither will be more accurate than the other.

The other issue, precision, is not investigated in this part of the study, but perhaps it is implied to be connected to the magnitudes of the integrand. Precision can be examined by computing the expected variance of the averages. One might anticipate that a larger integrand (such as found for CCoP) would have a larger variance, but my own (quick, not thoroughly checked) calculations indicate that the variance of the averages for both methods are exactly the same, and equal to (x1-x2)^2. Thus, there is no advantage to optimal transport in this respect either. It averages to zero for every lambda, but its fluctuations about zero are the same as the fluctuations in CCoP.

The final third of the manuscript begins to address the question of how the proposed method would be used in practice. The protocol laid out is really quite complicated. I must confess that I do not understand the instructions here completely:

a. Generate histograms for the endpoints. What variables am I histogramming against? The introduction indicates x, y as configurational coordinates, but how do I choose these? The examples (the shifted well and the single-->double well) involve a single, obvious coordinate, but in practical cases what would this be? It depends on details of the problem, of course, and this gap argues for having a more realistic example. Other methods for defining a path don't have to contend with this issue.

b. For some set of values of lambda in (0, 1), compute for each the Barycenter, which serves as the potential for each lambda. This involves computing an optimum p(x) by minimizing a sum of two Wasserstein distances. This is a formidable task, all the more so if dealing with multidimensional coordinates x.

c. Train a neural network for each lambda; my understanding is this is to allow extrapolation of the potential U_w(x;lambda) outside the region where it is sampled. Is this reliable for realistic applications?

d. Sample the learned potentials along the path from lambda = 0 to 1, applying BAR to compute each incremental free-energy difference. Why do the authors not consider MBAR (Shirts and Chodera, 10.1063/1.2978177) for processing their averages? It is designed to optimally compute free energies from multistage samples of the type performed here.

The single-->double-well toy model is investigated with isothermal molecular dynamics simulation. There is a single particle in this system. To add some slight degree of realism, the authors may wish to apply this using multiple non-interacting particles in the wells, which will give a bit more fluctuation and difficulty to the calculation, while still being relatively simple to treat. See the multi-harmonic model used, for example, in Schultz and Kofke, 10.1080/08927022.2020.1758695.

Figure 3 should indicate the value of lambda for each curve.

It is necessary to provide uncertainties on the data presented in Fig. 4 (and quoted in the text). Each calculation should be performed multiple times and uncertainties generated from the variance in the results.

The question of improved paths for free-energy perturbation was examined also by Lu et al (10.1103/PhysRevE.69.057702).

In summary, the most compelling argument for optimal transport is its potential to formulate a path that has reduced free-energy barriers. This is demonstrated in one, maybe two, toy models. Still, it is not shown that the protocol yields results that are more accurate or precise than alternatives. Reducing the barrier in and of itself may, but doesn't necessarily, lead to improved calculations. The method appears to be very daunting to implement in practice, certainly much more difficult than the methods currently in use. Lacking a persuasive demonstration of its effectiveness, I cannot recommend it for publication in JPC Lett.

Reviewer: 2

Comments to the Author

This is an interesting article but it needs some revisions. I agree that conventional Hamiltonian morphing methods may tend to destroy and rebuild distributions, as stated by the authors. The authors then argue that their formulation based on optimal transport tends to more gently move and adapt probability mass. Fair enough. The formal part of the article (pages 2-3) is not particular transparent. It is hard to interpret the meaning of the equations. Equation 8 is confusing because it is missing an equal sign. The authors are honest, as they point out that the optimal transport gentleness is not for free. As laid out on page 11, the suggested protocol requires (in step 3) to get the Wasserstein potential (9) for each lambda, and (in step 4) to train a neural network for each lambda such that one has an out-of-sample morphing Wasserstein potentials potential function. So, one has to train a neural network for a plain equilibrium free energy calculation? Are you sure your average users will be interested in doing this? Personally, I am skeptical.

The results shown in the paper are not entirely convincing. For example, in Figure 4 is shown the convergence of of a free energy calculation for a simple test system corresponding to a harmonic oscillator versus a shifted double-well potential. The double-well is zero centered whereas the harmonic oscillator is shifted of 0.5 nm, hence the systems do not bear configurational superposition. The authors argue that this test can be considered a prototypical case of two systems where there is no configurational overlap hence doing a perturbation from one Hamiltonian to another can be a hard task. In Figure 4, the results show that the convergence of the linear interpolation and Optimal Transport are almost identical! Furthermore, it is stated that the linear interpolation and linear interpolation plus minima correction give identical results (which is a bit surprising). The Minimum Variance Path (MVP) introduced by Blondel1 appears to perform poorly. Because this method does map the two end-states of the free energy transformation, it would be important to show that it does converge to the right answer ultimately (albeit more slowly). It this cannot be done, it suggests that maybe there is a bug somewhere. It would be nice to add also a comparison with the EDS (Enveloping Distribution Sampling) method since this is used by many people. Finally, Figure 4 would be a bit more informative by adding

error bars (estimated from multiple independent runs of the same number of steps). Lastly, the only illustrative examples are one-dimensional harmonic oscillators. How would you manage anything slightly more complicated, like an alchemical transformation of ethane into methanol for example?

Author's Response to Peer Review Comments:

Replies to Reviewers, manuscript jz-2022-035234

Dear Prof. Editor,

We are grateful for having given us the possibility to reply to Reviewers. We have answered below to the arisen issues. We now clarified some parts of the manuscript, performed additional experiments, and better underlined the value of the approach and of Optimal Transport (OT) in general as a novel asset for free energy computations.

Reviewer: 1

This manuscript describes optimal transport as a protocol for defining a path between two states of a system for the purpose of computing their free energy difference. The first third of the manuscript lays out the basic ideas and defines relevant quantities. I have the following minor suggestions to improve the clarity of this part

Thank you very much for the (minor) recommendations, which have helped improve the overall quality of our manuscript.

1. Near Eq. 3, C is defined as minimizing the cost "with respect to c(x,y)". The quantity being varied to find the minimum is gamma(x,y), so my understanding of "with respect to" in this context would be gamma, not c. I could see that "with respect to" might mean "based on the definition using", and arguably this is not incorrect usage. I think however it is confusing and I suggest the authors reconsider the phrasing of the statement if this is intended. Or perhaps they actually meant "with respect to gamma(x,y)", and this is just a typo.

Thanks. Yes, minimization is with respect to gamma(x,y) using c(x,y) as distance function. This has now been clarified in the revised manuscript.

2. The p appearing both as a superscript and subscript on W in Eq. 4 is confusing. It took me some time to understand that the superscript is an exponent, and the subscript indicates that it is a p-norm. I suggest putting the W and its arguments in brackets, with the p superscript on that, or putting brackets on the right-hand side with a 1/p exponent.

Thanks, we have added brackets.

3. After Eq. 4, it is stated that "one can obtain analytically the geodesic distribution". The context suggests this appears to be a consequence of the Wasserstein distance, but I don't see the connection.

This is more a consequence of the Gaussian distribution. In this very particular case, one can show there exists an analytical transport map, which morphs two Gaussians. In the manuscript, we wrote "for the specific case of Gaussian distributions and L2-norm, one can obtain…". The very first proof of the closed form Wasserstein distance for Gaussians was reported in 1982 (10.1016/0047-259X(82)90077-X).

What procedure does one perform to obtain the geodesic distribution? Is it gamma for the minimum when p1 and p2 are Gaussians? (apparently not, as it doesn't depend on y) Presumably, I could consult the reference for clarification, but I should be able to understand the statement by itself (apart from its derivation) without consulting another paper.

The analytical procedure is very peculiar for Gaussians only (unfortunately). In general, yes, one should minimize with respect to gamma(x,y), numerically. There exist approximate efficient algorithms, which work well on moderate dimensions such as the Sinkhorn algorithm from Cuturi.

4. If Eq. 8 is defining B, the barycenter, I suggest putting "B \equiv" on the left-hand side, so it is an equation. This makes it easier to understand when looking back at it from later in the paper.

Thanks. As noted also by the second Reviewer we have now improved notation.

The middle third of the paper treats a toy model, characterizing how the methods under consideration would perform when computing the free energy difference using thermodynamic
integration (TI). This part of the paper misses the mark, and does not provide support for the value of the optimal transport method. The performance of any free-energy calculation is concerned separately with the accuracy and the precision of the calculation. Regarding accuracy, assuming the system is well-sampled for each lambda (which is not an issue in this work), then the main determinant of accuracy is the curviness of the integrand -- more integration points are needed to navigate accurately where the integrand is not linear. An integrand the depends strictly linearly on lambda will have absolutely no difficulty yielding an accurate result (just two quadrature points is sufficient). Both optimal transport and the convex combination of potentials (CCoP) have integrands that depend linearly on lambda. It does not matter that one is horizontal and the other is not -- neither will be more accurate than the other.

We agree that for Gaussians the integrand for optimal transport and linear interpolation is linear. Yet the linear interpolation leads to big values of the integrand. This is not particularly amenable for finite-bits numerical representations. In general, having a limited numerical range in calculations is always a positive feature which should not be dismissed. We have now added some experiments that show the improved accuracy and precision of OT.

The other issue, precision, is not investigated in this part of the study, but perhaps it is implied to be connected to the magnitudes of the integrand. Precision can be examined by computing the expected variance of the averages. One might anticipate that a larger integrand (such as found for CCoP) would have a larger variance, but my own (quick, not thoroughly checked) calculations indicate that the variance of the averages for both methods are exactly the same, and equal to (x1-x2)^2. Thus, there is no advantage to optimal transport in this respect either. It averages to zero for every lambda, but its fluctuations about zero are the same as the fluctuations in CCoP.

Thanks for the observation. To estimate the variance of the calculations we run 3 replicas. Also, we run more simulations with a limited sample size to check the behavior of the standard deviation in the small sample regime. Besides reducing barriers, results now show that OT is both more accurate and more precise particularly at small sample sizes. In detail, MVP is precise but not accurate. Linear interpolation is accurate but not precise for small sample sets. Also, a nice feature, at least observed in this case for OT, is that the sign of the free energy difference is always correct, whereas in MVP and linear interpolation the sign is wrong at the beginning (small sample regime) and gets lately correct.

The final third of the manuscript begins to address the question of how the proposed method would be used in practice. The protocol laid out is really quite complicated. I must confess that I do not understand the instructions here completely:
a. Generate histograms for the endpoints. What variables am I histogramming against? The introduction indicates x, y as configurational coordinates, but how do I choose these? The examples (the shifted well and the single-->double well) involve a single, obvious coordinate, but in practical cases what would this be? It depends on details of the problem, of course, and this gap argues for having a more realistic example. Other methods for defining a path don't have to contend with this issue.
b. For some set of values of lambda in (0, 1), compute for each the Barycenter, which serves as the potential for each lambda. This involves computing an optimum p(x) by minimizing a sum of two Wasserstein distances. This is a formidable task, all the more so if dealing with multidimensional coordinates x.

We termed the protocol "illustrative" to show that the OT principle is rather powerful. We don't pretend at this stage to apply immediately this protocol to high dimensional problems. This will be the subject matter of future, extensive investigations. Indeed, possibly the OT principle could be embedded in an approximate way in more scalable methods. For instance, leveraging TFEP via normalizing flow with OT regularization could be a possibility. We have now modified the text accordingly.

c. Train a neural network for each lambda; my understanding is this is to allow extrapolation of the potential U_w(x;lambda) outside the region where it is sampled. Is this reliable for realistic applications?

Yes, we use the neural network to approximate the potential and interpolate. Neural networks have largely shown in the literate to be suitable for approximating potentials accurately (starting from the pioneering work by Behler and Parrinello). Training neural networks for free energy computations is a strategy already pursued by other groups (e.g., 10.1063/5.0018903).

d. Sample the learned potentials along the path from lambda = 0 to 1, applying BAR to compute each incremental free-energy difference. Why do the authors not consider MBAR (Shirts and Chodera, 10.1063/1.2978177) for processing their averages? It is designed to optimally compute free energies from multistage samples of the type performed here.

We are aware of MBAR but, in our experience, it does not contribute to significant improvements with respect to plain BAR applied pairwise with well converged sampling.

The single-->double-well toy model is investigated with isothermal molecular dynamics simulation. There is a single particle in this system. To add some slight degree of realism, the authors may wish to apply this using multiple non-interacting particles in the wells, which will give a bit more fluctuation and difficulty to the calculation, while still being relatively simple to treat. See the multi-harmonic model used, for example, in Schultz and Kofke, 10.1080/08927022.2020.1758695.

Thanks for the suggestion. We now added a series of simulations where we still employ the harmonic/double-well pair but employing 2 and 4 particles. Results in this new setting confirm and strengthen the findings for one single particle.

Figure 3 should indicate the value of lambda for each curve.

Thanks. We have added the lambda information to each curve.

It is necessary to provide uncertainties on the data presented in Fig. 4 (and quoted in the text). Each calculation should be performed multiple times and uncertainties generated from the variance in the results. The question of improved paths for free-energy perturbation was examined also by Lu et al (10.1103/PhysRevE.69.057702).

Thanks. We have repeated 3 times the computations. Error bars are now present in Figures 4 and 5. In Figure 5, they are minimally visible because we are using the longest simulations and hence variability is very low. Thanks a lot for this observation, now plot 4 and table 1 show the superior precision and accuracy of OT. We have added to the text the reference 10.1103/PhysRevE.69.057702 when discussing the results and the role of barriers.

In summary, the most compelling argument for optimal transport is its potential to formulate a path that has reduced free-energy barriers. This is demonstrated in one, maybe two, toy models. Still, it is not shown that the protocol yields results that are more accurate or precise than alternatives. Reducing the barrier in and of itself may, but doesn't necessarily, lead to improved calculations. The method appears to be very daunting to implement in practice, certainly much more difficult than the methods currently in use. Lacking a persuasive demonstration of its effectiveness, I cannot recommend it for publication in JPC Lett.

Thanks for pointing this out. We now hope to have shown with the additional experiments that OT-based approaches hold great potential.

Reviewer: 2

This is an interesting article but it needs some revisions. I agree that conventional Hamiltonian morphing methods may tend to destroy and rebuild distributions, as stated by the authors. The authors then argue that their formulation based on optimal transport tends to more gently move and adapt probability mass. Fair enough.

Thanks for the positive comment.

The formal part of the article (pages 2-3) is not particular transparent. It is hard to interpret the meaning of the equations. Equation 8 is confusing because it is missing an equal sign.

Thanks for pointing this out. We have improved notation in that and in the subsequent equation.

The authors are honest, as they point out that the optimal transport gentleness is not for free. As laid out on page 11, the suggested protocol requires (in step 3) to get the Wasserstein potential (9) for each lambda, and (in step 4) to train a neural network for each lambda such that one has an out-of-sample morphing Wasserstein potentials potential function. So, one has to train a neural network for a plain equilibrium free energy calculation? Are you sure your average users will be interested in doing this? Personally, I am skeptical.

We agree it is not trivial. However, if one extends the approach to collective variables, then the approach is possible. Also please consider that here the neural network is used to prove the efficacy of the approach. More scalable algorithms will be the subject matter of future investigations in the field. That's the reason why we termed the protocol "illustrative".

The results shown in the paper are not entirely convincing. For example, in Figure 4 is shown the convergence of of a free energy calculation for a simple test system corresponding to a harmonic oscillator versus a shifted double-well potential. The double-well is zero centered whereas the harmonic oscillator is shifted of 0.5 nm, hence the systems do not bear configurational superposition. The authors argue that this test can be considered a prototypical case of two systems where there is no configurational overlap hence doing a perturbation from one Hamiltonian to another can be a hard task. In Figure 4, the results show that the convergence of the linear interpolation and Optimal Transport are almost identical! Furthermore, it is stated that the linear interpolation and linear interpolation plus minima correction give identical results (which is a bit surprising). The Minimum Variance Path (MVP) introduced by Blondel1 appears to perform poorly. Because this method does map the two end-states of the free energy transformation, it would be important to show that it does converge to the right answer ultimately (albeit more slowly). It this cannot be done, it suggests that maybe there is a bug somewhere. It would be nice to add also a comparison with the EDS (Enveloping Distribution Sampling) method since this is used by many people. Finally, Figure 4 would be a bit more informative by adding error bars (estimated from multiple independent runs of the same number of steps).

Thanks for pointing these aspects out. These observations have significantly improved our findings. First, we have now repeated 3 times the same calculations randomizing the initial seed; this has allowed us to show error bars. Second, we have collected more simulations points. We have added 10 ns simulations (for each lambda value) and added short simulations (100 and 1000 samples). Now, Figure 4 shows that OT potentials are particularly effective both in terms of precision and accuracy in the small sample regime. We also repeated the computations for 2 and 4 particles strengthening the findings. We confirm that linear and linear corrected results are "nearly" identical when it comes to the final free energy value. This high reproducibility level is probably due to using 64 bits floating point numbers.
The original EDS method can be seen as a special case of MVP. In the EDS original paper (10.1063/1.2730508), Authors introduced a single bridging potential. Such a bridging potential is the MVP potential for lambda = 0.5 up to a constant. In our MVP computations we used more than one lambda value to maximize accuracy. Further developments of the EDS method allow setting a free parameter "s" (10.1063/1.2913050); our MVP tests correspond

hence to EDS using s=1 but using several bridging potentials (19 intermediate lambdas). We clarified this more in the text. Recently in the Riniker group (10.1021/acs.jcim.0c00520), it has been discussed the tight relationship between EDS and MVP and the convergence difficulties of these methods without a fine tuning of the free parameter "s". Note that OT has no free parameters to be set.

Lastly, the only illustrative examples are one-dimensional harmonic oscillators. How would you manage anything slightly more complicated, like an alchemical transformation of ethane into methanol for example?

We agree that it would be difficult to manage high dimensions using the current illustrative protocol. We have now clarified this more in the conclusions. The theory could be extended in principle to collective variables and the current protocol is chiefly "illustrative" as stated in the main text. To make optimal transport (OT) scalable is far from trivial. This will be the subject matter of intense investigations in the future. A first approach could be not to pretend to directly use OT but indirectly as a form of regularization. Indeed, the mentioned targeted FEP method assumes the existence of a map, and this can be delivered by normalizing flows. Here too, one is learning a neural network to run a free energy computation. Yet normalizing flows, per se, do not assure a smooth map. Hence, OT could come to the rescue by providing a suitable regularization operator. This regularization approach has been pioneered in machine learning (https://arxiv.org/abs/2006.00104), and it could be applied in free energy computations to create smooth and hence fast converging maps.

jz-2022-035234.R2

Name: Peer Review Information for "Optimal Transport for Free Energy Estimation"

Second Round of Reviewer Comments

Reviewer: 1

Comments to the Author

The authors have made a conscientious effort to respond to the reviewer comments, and I consider the revised manuscript suitably improved to be appropriate for publication in JPCLett. I have just minor revisions to request.

* The data listed in table 1 should be presented in a manner that is consistent with the uncertainties. There should be one, or at most two, digits given for the uncertainty, and the data values should include only the significant digits (as specified by the uncertainties). So for example

-0.246 +/- 0.278 should be -0.2 +/- 0.3

4.398 +/- 0.692 should be 4.4 +/- 0.7

-0.283 +/- 0.125 should be -0.28 +/- 0.13

etc.

* The confidence level of the uncertainties should be specified (e.g. 95%) An uncertainty based on 3 replicates requires a t-distribution, so for example for 95% uncertainty, the sample standard deviation would be multiplied by 4.3/sqrt(3) = 2.5 to give a 95% uncertainty (the sqrt(3) is to yield the standard deviation of the mean, and the 4.3 is the t-distribution multiplier for 2 DOF to encompass 95% of the probability).

Author's Response to Peer Review Comments:

As suggested we now report the results with the confidence level of 95%. Hence we modified the text, the table and figures 4,5 by replacing the standard deviation with the t-Student computed confidence interval (4.303 sigma)/sqrt(3). Also, we now show only two digits in the table and the manuscript.
